# Assessment of Lumbosacral Nerve Roots in Patients with Type 2 Diabetic Peripheral Neuropathy Using Diffusion Tensor Imaging

**DOI:** 10.3390/brainsci13050828

**Published:** 2023-05-21

**Authors:** He Chen, Yanyan Xu, Wei Wang, Ruifen Deng, Zhaoqing Li, Sheng Xie, Jinsong Jiao

**Affiliations:** 1 Department of Radiology, Peking University China-Japan Friendship School of Clinical Medicine, Yinghua Street 2, Chaoyang District, Beijing 100029, China; chenhe0911@163.com; 2 Department of Radiology, China-Japan Friendship Hospital, Yinghua Street 2, Chaoyang District, Beijing 100029, China; 3 Department of Neurology, China-Japan Friendship Hospital, Yinghua Street 2, Chaoyang District, Beijing 100029, China; 4 Department of Endocrinology, China-Japan Friendship Hospital, Yinghua Street 2, Chaoyang District, Beijing 100029, China

**Keywords:** diabetic peripheral neuropathy, lumbosacral nerve roots, magnetic resonance imaging, diffusion tensor imaging, tractography

## Abstract

Background: Diffusion tensor imaging (DTI) has found clinical applications in the evaluation of the central nervous system and has been extensively used to image peripheral neuropathy. However, few studies have focused on lumbosacral nerve root fiber damage in diabetic peripheral neuropathy (DPN). The aim of the study was to evaluate whether DTI of the lumbosacral nerve roots can be used to detect DPN. Methods: Thirty-two type 2 diabetic patients with DPN and thirty healthy controls (HCs) were investigated with a 3T MRI scanner. DTI with tractography of the L4, L5, and S1 nerve roots was performed. Anatomical fusion with the axial T2 sequences was used to provide correlating anatomical information. Mean fractional anisotropy (FA) and apparent diffusion coefficient (ADC) values were measured from tractography images and compared between groups. Diagnostic value was assessed using receiver operating characteristic (ROC) analysis. The Pearson correlation coefficient was used to explore the correlation between DTI parameters and clinical data and the nerve conduction study (NCS) in the DPN group. Results: In the DPN group, FA was decreased (*p* < 0.001) and ADC was increased (*p* < 0.001) compared with the values of the HC group. FA displayed the best diagnostic accuracy, with an area under the ROC curve of 0.716. ADC was positively correlated with HbA1c level (r = 0.379, *p* = 0.024) in the DPN group. Conclusions: DTI of lumbosacral nerve roots demonstrates appreciable diagnostic accuracy in patients with DPN.

## 1. Introduction

Diabetic peripheral neuropathy (DPN) is one of the most common and troublesome complications of diabetes [1,2], and it often remains undiagnosed until the later stages. As previously reported, more than half of diabetic patients could deteriorate to DPN [3], which creates a huge economic burden on both families and society [4]. DPN causes irreversible damage to the peripheral nerves [5]. Thus, early diagnosis is important in order to prevent the progression of DPN. Electrophysiology is recommended as the gold-standard tool to diagnose DPN [6], which can provide a reliable basis for estimating the speed and amplitude of motor nerve conduction [7]. However, electrophysiology is not able to detect focal neuropathy at an early and subclinical stage [8] and is time-consuming, invasive, and might be affected by many factors such as the proficiency and subjectivity of the operator [9]. Therefore, there is a need to find a convenient, reliable technique for the diagnosis of DPN.

Diffusion tensor imaging (DTI) is a noninvasive MR imaging modality that provides a quantitative insight into the neuronal architecture [10] and has been extensively used to image peripheral neuropathy [11,12,13,14]. Previous studies suggest that DTI may be the most sensitive noninvasive imaging method to detect microstructural alterations of peripheral nerves in DPN, and showed reduced fractional anisotropy (FA) of peripheral nerves in DPN patients compared with that of healthy controls (HCs) in the sciatic, tibial, and peroneal nerves [5,9,15,16,17]. However, distal nerves may not be the best site to detect diabetic nerve damage. Jende et al. found a proximal predominance of nerve lesions in DNP in T2-weighted imaging [18,19], while another literature source suggested that proximal nerve damage in diabetes parallels distal nerve function even before patients start to experience clinical symptoms [20]. Thus, for DPN patients, DTI of the lumbosacral nerve roots may be an alternative choice to detect neuropathy at an early stage.

The aim of the current study was to evaluate whether DTI of the lumbosacral nerve roots can be used to detect DPN. Furthermore, applying receiver operating characteristic (ROC) analyses, we aimed to determine the sensitivity and specificity of the MR methodology.

## 2. Materials and Methods

### 2.1. Subjects

Through public announcements, we recruited 32 patients with type 2 diabetes (8 females, 24 males, mean age 57.7 ± 10.1, age range 38–80, and median age 58.5) from the Department of Endocrinology and Neurology (China-Japan Friendship Hospital) and 30 healthy control subjects (18 females, 12 males, mean age 49.6 ± 17.3, age range 19–79, and median age 46). All participants were examined between June 2021 and January 2022. All participants signed informed consent forms, and the Institutional Review Board of China-Japan Friendship Hospital approved the study (approval No. 2021-89-K54).

First, the diagnosis of diabetes was in accordance with the standards of The American Diabetes Association [21]. Then, the diagnosis of DPN in diabetic patients was determined according to the established clinical protocol, which requires the presence of more than one symptom (numbness, weakness, prickling, burning, or aching pain) or signs in keeping with a distal symmetrical neuropathic pattern of onset and progression (abnormal knee or ankle reflexes, light touch, monofilament, temperature, or vibration sensation) and abnormal nerve conduction study [22].

The exclusion criteria were: age <18, other neurological or endocrine disorders, musculoskeletal disorders, metabolic dysregulation, malignant or infectious diseases, any history of lumbar trauma or surgery, and contraindications to MRI. HCs answered the Michigan Neuropathy Screening Instrument (MNSI) questionnaire to exclude symptoms of neuropathy and diabetes [23], and the blood glucose level was normal within half a year.

### 2.2. Electrophysiological Examination

The nerve conduction study (NCS) was performed using conventional surface electrode techniques at a skin temperature of 31–33 °C by an experienced neurologist with EMG equipment (Keypoint v. 2.11, Dantec, Skovlunde, Denmark). The room was kept quiet and warm during the examination, and the patients remained in a relaxed state. Motor and sensory NCSs were performed in the tibial and median nerves, and the nerve conduction velocities (NCVs) and compound muscle action potential (CMAP) amplitude were determined. The results were compared with the published values in Preston and Shapiro’s work [24], and the presence of neuropathy was based on abnormal values in at least two nerves.

### 2.3. MR Examination

The lumbosacral region of all subjects was examined using a 3.0 T MR system (Inginia 3.0 T, Philips Medical Systems, Best, The Netherlands) with a 32-channel body coil employed. All subjects underwent imaging in the supine position with arms alongside the body and head first. Each scanning session comprised a DTI sequence (TE 78 ms, TR 2700 ms, flip angle 90°, slice thickness 5 mm, no interslice gap, number of slices 20, FOV 160 × 249 mm^2^, matrix size 108 × 165, number of signals acquired 1, b value 0 and 800 s/mm^2^, number of directions of motion-probing gradients 15, fat saturation SPIR, and acquisition time 5 min 32 s) and an axial T2W mDixon image (TE 100 ms, TR 1705 ms, flip angle 90°, slice thickness 5 mm, no interslice gap, number of slices 20, FOV 160 × 249 mm^2^, matrix size 200 × 288, number of signals acquired 1, and acquisition time 2 min 20 s; the same center position and orientation as those used with DTI were used) to provide correlating anatomical information.

MR sequences were centered on the lower edge of the L5 vertebra to ascertain that the anatomical region mapped with MR was comparable in all participants. All MR studies were performed by the same radiologist with more than 5 years of clinical experience in MR studies.

### 2.4. Image Postprocessing

**Figure 1:**
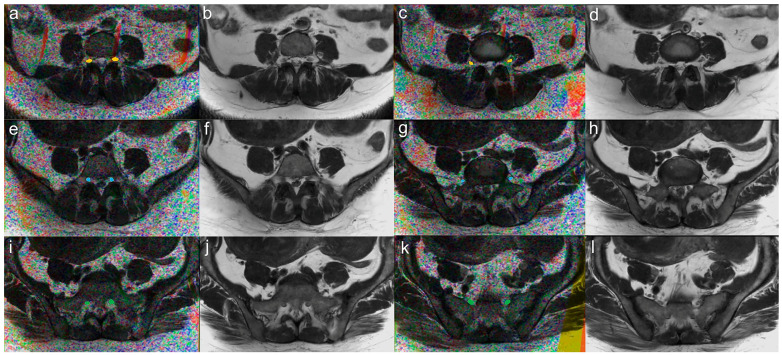
Axial view of fusion (**a**,**c**,**e**,**g**,**i**,**k**) and T2-weighted (**b**,**d**,**f**,**h**,**j**,**l**) images from a DPN patient (54-year-old female). ROI placement for measurement of ADC and FA. The ROIs of seeding points were placed at two levels of one nerve root in the fusion images: one was at the level of middle spinal body (**a**,**e**,**i**) and another was at the level of inferior spinal disc (**c**,**g**,**k**). Yellow ROIs represent the L4 nerve roots; blue ROIs represent the L5 nerve roots; and green ROIs represent the S1 nerve roots.

DTI data were transferred to the Extended MR Workspace workstation (R2.6.3.5 HF 3 2013, Philips Medical Systems, Best, The Netherlands) for further processing. T2W images were used for image fusion and anatomic correlation to ensure that only the nerve tissue was included in the region of interest (ROI), and the surrounding fat or tendon was excluded. Two experienced radiologists manually drew an ROI, respectively, blinded to each other. The ROIs of seeding points were placed at two levels of one nerve root: one was at the level of the middle spinal body, and another was at the level of the inferior spinal disc (Figure 1). When sketching the ROI, the area was selected as close to the nerve edge as possible while avoiding the muscles, fat, and other tissues around the nerve. Then, the fiber track processing was executed to obtain the fiber track color imaging of the L4, L5, and S1 nerve roots, and the FA and apparent diffusion coefficient (ADC) values of each nerve root were calculated. The following parameters were used for fiber track processing: FA threshold 0.15, minimum fiber length 10 mm (smaller fibers were excluded), and smoothness 27. These parameters were chosen in order to exclude as many extra nerve root voxels as possible. The measurements of the FA and ADC values were conducted by two radiologists independently, and the average of the two radiologists’ results were used for further analysis.

### 2.5. Statistical Analysis

Statistical analyses were performed using SPSS (version 22, IBM Inc., Chicago, IL, USA). All data from HCs and patients were described by using mean and standard deviation. The inter-reader agreement of the FA and ADC values was assessed using intraclass correlation coefficient (ICC) analysis, and an ICC above 0.75 was defined as good agreement. The data were tested for normal distribution using the Kolmogorov–Smirnov test. An independent samples t-test was used to compare the mean FA and ADC values between the HC and DPN groups. The diagnostic accuracy of DTI for DPN was evaluated with receiver operating characteristic (ROC) analysis, and the area under the curve (AUC), cut-off values, sensitivity, and specificity were calculated. The Pearson correlation coefficient was used to explore the correlation between DTI parameters and clinical data and NCS. *p* < 0.05 was considered statistically significant.

## 3. Results

### 3.1. Demographic and Clinical Data

There was no difference between the two groups for participants’ age (*p* = 0.085) and body mass index (BMI, *p* = 0.114). There was a significant difference in sex (*p* = 0.005) between these two groups; however, Simon et al. [12] found no significant changes between genders in the diffusion metrics of the peripheral nerves, so this difference will not affect the accuracy of the results. The demographic results and clinical data are presented in Table 1.

### 3.2. Removal of Compressed Nerve Roots

Several studies have shown that there are significant changes in FA and ADC values in the compressed lumbosacral nerve roots [25,26]; this change is similar to the expected result of DTI in DPN and could affect the accuracy of the results. The T2W images provided a nice depiction of the L4, L5, and S1 nerve roots. Two experienced radiologists, blinded to each other, recognized the compressed nerve roots on the T2W images, respectively, and distinguished 22 nerve roots compressed by lumbosacral disc herniation from 372 nerve roots (11 for each group). All the 22 nerve roots were not included in the subsequent statistical analysis.

### 3.3. Tractography and DTI Metrics

**Figure 2:**
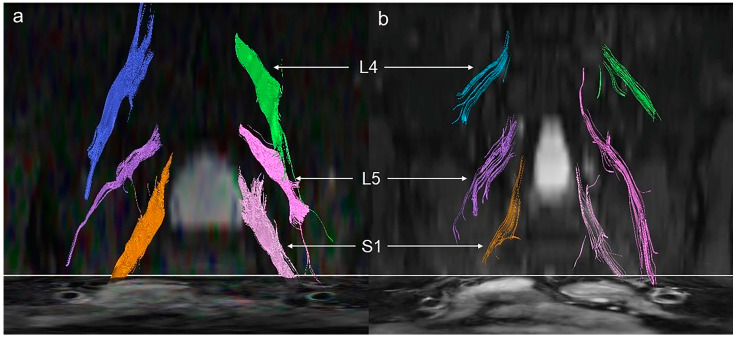
Lumbosacral nerve root fiber track. Fiber track of L4, L5, and S1 nerve roots is shown in different colors. (**a**) 55-year-old female HC. (**b**) 54-year-old female type 2 diabetes patient. The following parameters were used for fiber track processing: FA threshold 0.15, minimum fiber length 10 mm (smaller fibers were excluded), and smoothness 27.

An axial view of fusion and the T2-weighted images is shown in Figure 1. Representative lumbosacral nerve root fiber track of the two groups can be found in Figure 2. It can be seen that fiber track in the control patient are dense (Figure 2a), which decreases for the DPN patients (Figure 2b).

**Figure 3:**
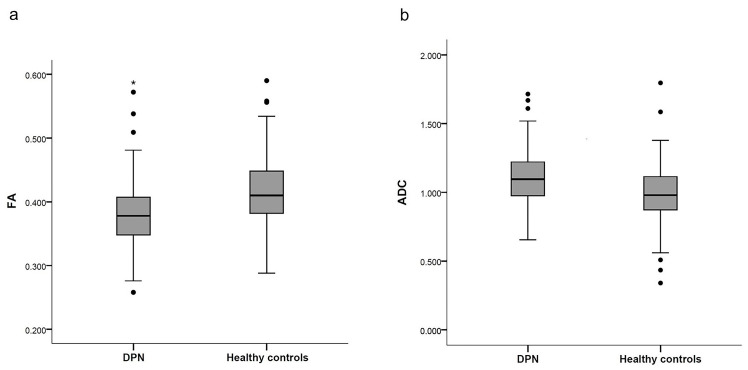
Box plots of DTI metrics in HC and DPN groups. FA values (**a**) were significant lower in DPN than in HCs (*p* < 0.001); ADC values (**b**) were higher in DPN than in HCs (*p* < 0.001). FA values have no unit; ADC values are 10^−3^ mm^2^/s. The plots illustrate the 25th and 75th percentiles (boxes), adjacent values (asterisk), outliers (dots), and median values of the groups (black horizontal lines in grey boxes).

The inter-reader agreements for all DTI metrics were good (shown in Table 2). The DTI metrics’ values of the lumbosacral nerve roots in the HC and DPN groups are presented in Table 3 and Figure 3. In the DPN group, FA was decreased and ADC was increased compared with the values of the HC group (*p* < 0.001).

### 3.4. Evaluation of Diagnostic Performance

**Figure 4:**
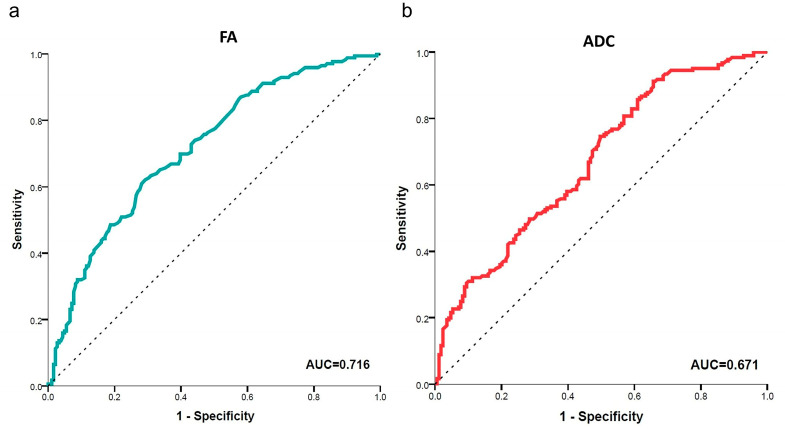
ROC curves of the sensitivity and specificity of FA (**a**) and ADC (**b**) values in the lumbosacral nerve roots. The areas under the curves (AUC) are shown in the bottom right corner. FA had the best performance for diagnosing DPN (AUC values of FA is 0.716).

The ROC curves for both FA and ADC are shown in Figure 4. The cut-off value obtained with the ROC analysis was 0.3995 for the FA values (sensitivity, 72.4%; specificity, 65.1%; and AUC, 0.716) and 0.894 × 10^−3^ mm^2^/s for the ADC values measurement (sensitivity, 91.7%; specificity, 34.3%; and AUC, 0.671).

### 3.5. Correlation between DTI Parameters and Clinical Data and NCS

The correlation between the DTI parameters and the clinical data and NCS in the DPN group was explored. ADC was positively correlated with the HbA1c level in DPN patients (*r* = 0.379, *p* = 0.024), while no significant correlation between ADC and diabetes duration was found. There was no correlation between FA and HbA1c level or diabetes duration. There were no significant correlations between DTI parameters and neurophysiological parameters. However, there was a tendency of correlation between FA and CMAP in the right lower limb (*r* = 0.304, *p* = 0.090).

## 4. Discussion

In the present study, we demonstrated that DPN patients had decreased FA and an increased ADC of the lumbosacral nerve roots compared with the values measured in the HCs. The DTI findings in this study indicated that diffusion in the lumbosacral nerve roots became more isotropic because of nerve injury in DPN patients. It reflected the pathophysiological changes of DPN, including microcirculatory changes, demyelination of the nerve fibers, and axon degeneration [27]. Moreover, we discovered that the ADCs were significantly correlated with glycated hemoglobin (HbA1c) levels in DPN patients, one of the major predictors of diabetic neuropathy [28].

Previous studies have found significant changes in the diffusion properties of the sciatic and tibial nerves, as well as the tibial nerve and peroneal nerve in DPN patients [5,15,16]. FA is a metric of anisotropy, which reflects the integrity of nerves [5], and its value is mainly altered by the density and diameter of the axon and the density and thickness of the myelin sheath [29]. When there is axonal damage, the FA value is reduced [30]. The ADC reflects the diffusion speed of the water molecules along the nerve fibers, and accordingly, demyelination can lead to an increased ADC [31]. Although diabetic neuropathy is not considered primarily a demyelinating neuropathy, Schwann cells are targeted by chronic hyperglycemia, and more severe cases of diabetic neuropathy in patients include features of demyelination [32,33]. Additionally, Schwann cell damage might lead to several alterations in the axons [34,35]. Therefore, the abnormal diffusion parameters in our studies indicate diabetes-mediated peripheral nerve damage.

The ROC curve shows that among all DTI metrics, FA was the most accurate index for diagnosing DPN (the AUC value of FA is 0.716), which was close to the findings of Wu et al. [9]. Experimental studies have shown that FA correlates more strongly with axonal density [36,37], and ADC changes in relation to myelin density and myelin thickness [31]. In DPN, the progressive loss of axons is considered more prevalent than demyelination [38,39], suggesting that FA has the best discriminatory performance compared with the ADC in DPN patients. Marianna et al. [14] had the same observations in carpal tunnel syndrome (CTS) patients, and they thought the ADC was an isotropic value that assesses the diffusion of water molecules in all directions, whereas FA assesses diffusion in only one preferential direction; this is why FA is a more reliable parameter than the ADC.

It is noteworthy that in our studies, we measured the diffusion metrics of the lumbosacral nerve roots, instead of the more distal peripheral nerves as in previous studies. There are several technical advantages for the diffusion metrics derived from the tracked fibers. First, image qualities for the DTI of the lumbosacral nerve roots are better than those for the distal nerves; this is mainly due to lumbosacral nerve roots being relatively linear in this location, and movement artifacts can be kept to a minimum. Second, the lumbosacral nerve roots are less affected by vessel motion than the tibial and common peroneal nerves. Third, measurements for the lumbosacral nerve roots are more reliable because of the larger diameters and the large extent of peripheral fat, which makes it easier to detect and draw ROIs. The ICC for the diffusion metrics ranged from 0.847 to 0.934 in our study, which is higher than that in previous studies [9,15]. A previous study also found high ICC reliability for the sciatic nerve, but a lower one for the tibial nerve in type 1 diabetes [15].

Considering diabetic neuropathy is a unique neurodegenerative disorder of the peripheral nervous system, it seems that investigation of distal nerves such as tibial and common peroneal nerves is the first choice. However, substantial experimental evidence supports the idea that the entire neuron, from the perikaryon to the terminal, is targeted by diabetes [20,40]. It is thought that changes in axons, especially distal terminals, are associated with changes in the neuronal perikaryal [2]. Indeed, sensory neurons within the dorsal root ganglia alter their phenotype in chronic experimental diabetes, which might be critical in how they support distal axon branches [41,42]. Some believe that diabetic damage first targets the neuron perikaryal that reside in the dorsal root ganglia and act to support the axons instead of peripheral axons and their associated Schwann cells [43]. From this view, the detection of neural damage by measuring lumbosacral nerve roots which include dorsal root ganglia may be a better choice. Some studies found a reduction in FA both proximally and distally [15,20], while Vaeggemose et al. [5] even found that the abnormalities were more pronounced at the proximal level than at the distal level.

Some previous studies found a relationship between DTI metrics and electrophysiological parameters [5,9,17]. Our results suggested a tendency of correlation between FA and CMAP in the right lower limb (r = 0.304, *p* = 0.090). This evidence indicates that DTI metrics can be used to reflect diabetic-induced peripheral nerve damage. Furthermore, we evaluated the relationship between DTI metrics and clinical data, and found that ADCs were significantly correlated with HbA1c levels in DPN patients. Hyperglycemia can cause the demyelination of the nerves and subsequent axonal degeneration, which is one of the mechanisms of DPN [44]. Alterations of myelin density and myelin thickness lead to changes in ADCs [31].

Our study suggested that FA was an accurate index for diagnosing DPN and the ADC is a sensitive index to identify the presence of DPN. Due to the invasiveness of electrophysiological examination, MR DTI scanning of lumbosacral nerve roots may be a more convenient technique to diagnose DPN. A previous prospective study suggested that DTI can detect subclinical ulnar neuropathy at the elbow, which adds diagnostic value as a highly sensitive technique for the detection of peripheral neuropathy [11]. DTI was also proved to be able to identify potential abnormalities of brain white matter in T2DM patients without cognitive complaints [45]. However, whether DTI can provide an earlier diagnostic tool than electrophysiological examination for DPN remains to be further investigated.

The DTI procedure was performed by using spin echo EPI and sensitivity encoding (SENSE). SENSE greatly enhances the quality of diffusion-weighted EPI by reducing blurring and off-resonance artifacts [46]. All MR studies were performed by the same radiologist with more than 5 years of clinical experience in MR studies to ensure the comparable anatomical region was mapped in all participants. The fiber track technique is quite operator-dependent, and the operator should have detailed knowledge of the neuroanatomy, ROI location, and placement. The measurements of the FA and ADC values were conducted by two radiologists independently, the reliability of the method was determined by ICC, and ICC coefficients for FA and ADC values were both above 0.85. ICC values exceeding 0.75 indicate DTI to be a reliable MR method for detecting peripheral nerve lesions. In addition, DTI has a lower spatial resolution and is sensitive to magnetic susceptibility artifacts and chemical shift artifacts, which increase the difficulty and inaccuracy of measurement. SE-EPI technology improved the signal-to-noise ratio and the image quality, and shortened acquisition time significantly. Moreover, an axial T2W image (the same center position and orientation as DTI was used) was acquired to provide correlating anatomical information. T2W images are sensitive to edema and fat. We applied a strong fat saturation pulse to remove the epineural fat signal adjacent to the nerve fascicles, causing fat and connective tissue to appear dark in the MRIs. We could draw ROIs precisely in the DTI and T2W fusion images.

A number of limitations apply to this study. First, our sample size was relatively small and this study did not include type 2 diabetes patients without DPN. Therefore, whether DTI can differentiate DPN patients from type 2 diabetes patients without DPN cannot be ascertained and we need a further comparative study to test the hypothesis. A prospective, longitudinal study will be better for evaluation of the technique. Second, it is important to assess the impact of systematic errors on the DTI measurements because sometimes it can be large. Factors such as gradient coil inhomogeneities and motion artifacts have been identified as significant sources of error. We did not address the issue of proposing a curvilinear space with constant magnetic field gradients, which could be addressed by the generalized Stejskal–Tanner equation for nonuniform gradients [47]. Meanwhile, nonuniformities in magnetic field gradients can cause serious artifacts and potential errors in diffusion imaging [48]. Applying a correction method to address these errors has the potential to enhance the accuracy and reliability of DTI-based tractography [49]. Additionally, the BSD-DTI calibration technique [50] holds promise as an approach to mitigate these errors and further improve the quality of DTI data. It was proposed to use a curvilinear space in which the magnetic field gradients are constant. To improve the accuracy of DTI measurements, advanced acquisition schemes, such as multishell or high-angular-resolution diffusion imaging, are preferred to obtain more precise and detailed information about tissue microstructures. Additionally, emerging technologies, such as novel gradient waveform designs and advanced reconstruction algorithms, show potential for improving the accuracy and reliability of DTI measurements. Moreover, advanced post-processing techniques, including advanced tensor modeling and diffusion-model-based analysis, may be applied to obtain more accurate and robust estimates of diffusion parameters. In future research, the validation of DTI measurements could be conducted through phantom studies or by employing computer simulations to assess the systematic errors induced by diffusion gradient inhomogeneity [51]. Such validation approaches would not only enable the assessment of measurement accuracy but also have significant implications for the interpretation of DTI-based research findings. Third, because electrophysiological examination was performed only in the right lower limb for some patients, we lack some electrophysiological data for the analysis of the left lower limbs.

## 5. Conclusions

In summary, this study showed that peripheral nerve damage can be detected in the lumbosacral nerve roots using DTI in patients with DPN. FA demonstrated a tendency of correlation with the impairment of nerve conduction parameters, while ADC exhibited a positive correlation with HbA1c levels in the DPN group. DTI of lumbosacral nerve roots has several advantages in the detection of peripheral nerve dysfunction. It may be a promising tool in the early detection of DPN in diabetic patients.

## Figures and Tables

**Table 1:** Demographic and clinical data of different groups.

Variables	HCs (*n* = 30)	DPN (*n* = 32)	*p*
Age (years)	49.57 ± 17.29	57.72 ± 10.08	0.085
Sex (female/male)	18:12	8:24	0.005
BMI (kg/m^2^)	23.48 ± 3.48	24.87 ± 3.44	0.114
Diabetes duration (years)	-	15.03 ± 8.14	-
FBG (mmol/L)	-	9.04 ± 3.07	-
PBG (mmol/L)	-	18.21 ± 5.08	-
HbA1c (%)	-	8.31 ± 1.81	-
Fasting insulin (μIU/mL)	-	8.83 ± 4.60	-
Fasting C-Peptide (ng/mL)	-	1.82 ± 1.02	-
IAA (COI)	-	1.21 ± 2.01	-
Urine glucose (mmol/L)	-	24.71 ± 26.23	-
Right tibial NCV (m/s) *	-	39.82 ± 5.01	-
Left tibial NCV (m/s) *	-	39.88 ± 4.76	-
Right tibial CMAP (mV) **	-	6.21 ± 4.42	-
Left tibial CMAP (mV) **	-	5.03 ± 3.03	-

Notes: Values are presented as number or mean ± standard deviation, * *n* = 32, ** *n* = 27. HCs: healthy controls; DPN: diabetic peripheral neuropathy; BMI: body mass index; FBG: fasting blood glucose; PBG: postprandial blood glucose; HbA1c: glycated hemoglobin; IAA: insulin autoantibody; NCV: nerve conduction velocity; CMAP: compound muscle action potential.

**Table 2:** The inter-reader agreement in diffusion tensor imaging analyses.

	L4N		L5N		S1N	
	ICC	95%CI	ICC	95%CI	ICC	95%CI
FA	0.905	0.863–0.934	0.899	0.858–0.929	0.891	0.849–0.923
ADC	0.893	0.847–0.926	0.885	0.840–0.919	0.897	0.856–0.926

ICC: intraclass coefficient; CI: confidence interval; FA: fractional anisotropy; ADC: apparent diffusion coefficient; L4N: L4 nerve roots; L5N: L5 nerve roots; S1N: S1 nerve roots.

**Table 3:** Comparison of the DTI metric values within groups.

Variables	HCs	DPN	*p*	*t*	*F*
FA	0.419 ± 0.052	0.380 ± 0.049	<0.001	−7.072	1.440
ADC (10^−3^ mm^2^/S)	0.985 ± 0.198	1.107 ± 0.189	<0.001	5.879	0.228

Values are presented as mean± standard deviation. HCs: healthy controls; DPN: diabetic peripheral neuropathy; FA: fractional anisotropy; ADC: apparent diffusion coefficient.

## Data Availability

The datasets generated during the current study are available from the corresponding author upon reasonable request.
